# Tumour Hidden behind Thoracic Spine Pain: A Rare Case of Neuroblastoma in a Young Mother—A Case Report

**DOI:** 10.3390/ijerph192013448

**Published:** 2022-10-18

**Authors:** Valerio Passudetti, Luca De Leo, Filippo Maselli, Raffaello Pellegrino, Fabrizio Brindisino

**Affiliations:** 1 Department of Clinical Sciences and Translational Medicine, Medicine and Surgery School, University of Roma “Tor Vergata”, 00133 Rome, Italy; 2 Check-Up Center Private Practice, 73020 Lecce, Italy; 3 Department of Human Neurosciences, University of Roma “Sapienza”, 00185 Rome, Italy; 4 Sovrintendenza Sanitaria Regionale Puglia INAIL, 70126 Bari, Italy; 5 Antalgic Mini-Invasive and Rehab-Outpatients Unit, Department of Medicine and Science of Aging, University “G. d’Annunzio” Chieti-Pescara, 66100 Chieti, Italy or; 6 Department of Medicine and Health Science “Vincenzo Tiberio”, University of Molise C/da Tappino c/o Cardarelli Hospital, 86100 Campobasso, Italy

**Keywords:** differential diagnosis, referral and consultation, physical therapists, oncology, back pain

## Abstract

Background: Neuroblastoma (NB) is the most common form of paediatric malignancy, responsible for up to 15% of cancer deaths in children, whereas in adults, its onset is a rarer event, despite being characterized by greater lethality. The purpose of this case report was to describe the clinical presentation, physical examination, and clinical decision-making process in a patient with Neuroblastoma mimicking thoracic spine pain of musculoskeletal origin. Methods: a thirty-two-year-old mother complained of thoracic spine pain on her left vertebral side and in her left periscapular muscles; her pain was constant, deep, and worse at night; she also experienced pain during physical exertion of her upper limbs; the patient also reported pain in her left breast. Results: the physiotherapist’s anamnesis and physical examination led him to suspect the need for an extra-expertise pathology and to refer his patient to another medical specialist; the subsequent investigations revealed a poorly differentiated Neuroblastoma ALK + (IIC) in the posterior mediastinum on the left; the patient underwent surgery excision after 4 months. Conclusions: differential screening should be a physiotherapist’s fundamental skill in their patients’ clinical management, especially in direct access cases; the physiotherapist has an ethical and moral duty to conduct differential screening, in order to rule out extra-expertise pathologies—both when patients self-refer for rehabilitation assessment, and when they are referred by other practitioners.

## 1. Introduction

Neuroblastoma (NB) is the most common form of paediatric malignancy. It represents approximately 10% of all childhood cancers, and is responsible for up to 15% of cancer deaths in children [1]. It is an extra-cranial solid tumour, which occurs sporadically [2] or, more rarely (1–2%), has a familiarity [2].

The prevalence of NB is about 1 case per 7000 live births, and its annual incidence has risen to 700 new cases in the United States [3]. Its average age of diagnosis is 18 months, and it affects children under the age of 10 in 90% of cases [4]. Neuroblastoma is extremely rare in the adult population (0.2 cases per million), whilst also having a greater lethality [5] (up to 71% of cases) [6,7]. Moreover, in this clinical case, the NB presented a particular histology of a poorly differentiated subtype, which increased the rarity of the episode. Furthermore, in Italy, only four or five cases of NB are reported each year [6].

Neuroblastoma mainly originates in the adrenal medulla, which is the most common primary site in about half of the cases (47%), followed by the abdomen and retroperitoneal structures (24%), the thorax (15%), the neck (3%) and the pelvis (3%) [7]. A peculiar feature of NB is its high frequency of metastatic disease at diagnosis [3]—metastasis is predominantly found in the bone marrow or cortical bone, the distal and regional lymph node stations and liver, the lungs, the central nervous system, and the dermis and epidermis [7,8], regardless of the primary origin. To date, there are no specific treatment guidelines for adult patients with NB, despite its tumour biological differences and clinical manifestations compared to those present in younger subjects—i.e., the presence of n-MYC gene alterations, which are hardly found in adult patients, or the release of urinary catecholamines, also rarer in more mature subjects [9,10]. Therefore, due to the lack of specific recommendations, adults are generally treated according to international paediatric guidelines [11]. Other differences which contribute to differentiate adult neuroblastoma from younger patients include the prevalence of ganglioneuroblastoma (the nodular variant) in the localized resectable tumours, the infrequency of elevated values of catecholamine metabolites in urine, and the infrequency of abnormalities of the MYCN gene and 1p chromosome [6]. Moreover, in <2% of cases diagnosed in adolescence, the tumour is characterized by poor chemosensitivity, an indolent course, and a worse outcome than in the younger population [12,13].

Futhermore, in adult tumours, the biological abnormalities typically associated with an unfavourable clinical course in younger NB patients are quite infrequent, thus suggesting that in older patients there are different genes influencing the tumour’s ability to respond to therapy [14]. This would account for the peculiar clinical behaviour of these tumours in adults, particularly in cases of non-disseminated disease.

Moreover, NB has various clinical presentations, depending on locations and areas affected by its metastases [2]. Notably, some particular clinical manifestations, such as localized pain and difficulty or inability in physical efforts [2], can mimic a musculoskeletal disorder.

Neuroblastoma staging is performed using preoperative imaging, which identifies the presence/absence of “Image-Defined Risk Factors” (IDRFs), such as tumour infiltration into vital structures, such as arteries, veins, airways, the spinal canal, or organ parenchyma [15]. The main NB classification is the “International Neuroblastoma Risk Group Staging System—INRGSS” [16,17]. This classification model includes four categories (I1, I2, M and MS), and combines tumour histopathology with patients’ clinical and biological characteristics, such as age (less than 1.5 years, between 1.5 and 5 years, and over 5 years of age), Mitosis-Karyorrhexis Index (MKI), and presence of Schwann cells in the stroma. The diagnosis of NB is made through laboratory tests (blood and urine tests); Computed Tomography and Nuclear Magnetic Resonance for neoplasm in situ analysis; Metaiodobenzylguanidine Scintigraphy; positron emission tomography for metastasis identification; and biopsy for histological evaluation [18,19,20,21].

NB treatment strategies (Table 1) include simple observation, cytoreductive surgery, chemotherapy, radiotherapy, high-dose therapy (HDT) with autologous hematopoietic stem cell rescue, and immunotherapy [5].

The purpose of this study was to describe the signs and symptoms which led a physiotherapist to suggest the presence of an extra-professional pathology in an adult patient’s clinical presentation of chest and thoracic spine pain. It also describes the subsequent clinical screening processes, as well as laboratory and imaging tests that led to the NB diagnosis, the subsequent disease staging, its surgical resolution, and the patient’s rehabilitative follow-up.

## 2. Case Description

A thirty-two-year-old woman, an architect, visited to the authors’ rehabilitation clinic complaining of back pain. The patient had already suffered from back pain the year before, for which she had already resorted to the authors’ clinic. The patient’s symptom was easily evoked through the palpation of her periscapular muscles, and it disappeared after three sessions of manual therapy and therapeutic exercise.

Eventually, the patient reported a different kind of pain, describing her complaint as “bone pain” that was close to her dorsal spine; sometimes, she felt it exactly on her left vertebral side, while on other occasions it involved a more widespread area, including her left periscapular muscles. Her pain appeared progressively about one month before, and its intensity had risen over the weeks, until it reached a 6/10 level on the Numeric Pain Rating Scale (NPRS).

The patient described her pain as constant, sometimes quite superficial, and at other times more acute and deep (7/10 NPRS), resulting from upper limb efforts (i.e., wiping the floor or vacuuming).

At night, her pain worsened while in the left lateral decubitus position, (7/10 NPRS), forcing her to change positions to fall asleep, whereas lying in a prone position provided some pain relief (3/10 NPRS).

The patient also reported that, about two months earlier, she started to feel some discomfort in her left breast—intermittent and localized around her left nipple—especially after feeding, which had increased over time (5/10 NPRS), becoming constant, widespread, deep, and sharp from her chest to her back. This pain could not be evoked through a breast self-exam, and it worsened at night (7/10 NPRS), especially while in the left lateral decubitus position.

When asked about her family clinical history, the patient reported that her mother died of lung cancer. When asked about her previous medical history, the patient reported that, two years before, she had undergone a left breast benign lump removal, and that, about ten days prior to the physiotherapist’s examination, she had consulted her General Practitioner (GP) to report her left breast symptoms.

Her GP had performed the palpation of the breasts—looking for suspicious masses—and a breast ultrasound—both results came back negative. Subsequently, her GP prescribed paracetamol (1000 mg, once a day for 5 days) for suspected structural changes in the mammary ducts; this resulted in pain reduction, both in her breast and in her spine (4/10 NPRS both).

The physiotherapist noticed that the patient had lost weight, being thinner than she was during her previous year’s treatments: the patient reported having not followed any diet, and that she suffered from congenital hypothyroidism (which was under drug treatment); however, she realized she had not had much appetite in the recent months.

When asked about the possible presence of other associated symptoms, the patient replied that she felt tired and that after some efforts (i.e., going shopping or taking care her children) she felt exhausted. Furthermore, for about two months, the patient had been complaining of intermittent dyspnea—she found it difficult to breathe deeply, and she felt the urge to cough in order to get rid of this feeling of pressure and obstacle.

Lastly, the patient reported her spinal pain, breast pain, and wheezing were not necessarily concomitant, but sometimes her breast pain was evoked or made worse by her coughing or sneezing.

### 2.1. Physical Therapy Examination

The physiotherapist performed a comprehensive evaluation of the patient’s vital parameters [26,27], as well as active and passive thoracic ranges of motion [28]; moreover, provocative tests were administered, as detailed in Table 2. The anamnestic collection of red flags (Table 3) suggested an extra-professional pathology [29].

When questioned about her expectations regarding her clinical condition management, the patient wished for an improvement in her back pain, since it adversely affected her quality of life and her relationships with family and friends.

Finally, the physiotherapist administered the Italian version of the SF-36 (Table 1, baseline column) [30].

### 2.2. Referral, Diagnostic Imaging and Surgery

The physiotherapist referred the patient to her GP for a second evaluation through a detailed letter, describing the main features of her clinical presentation and physical examination.

**Figure 1:**
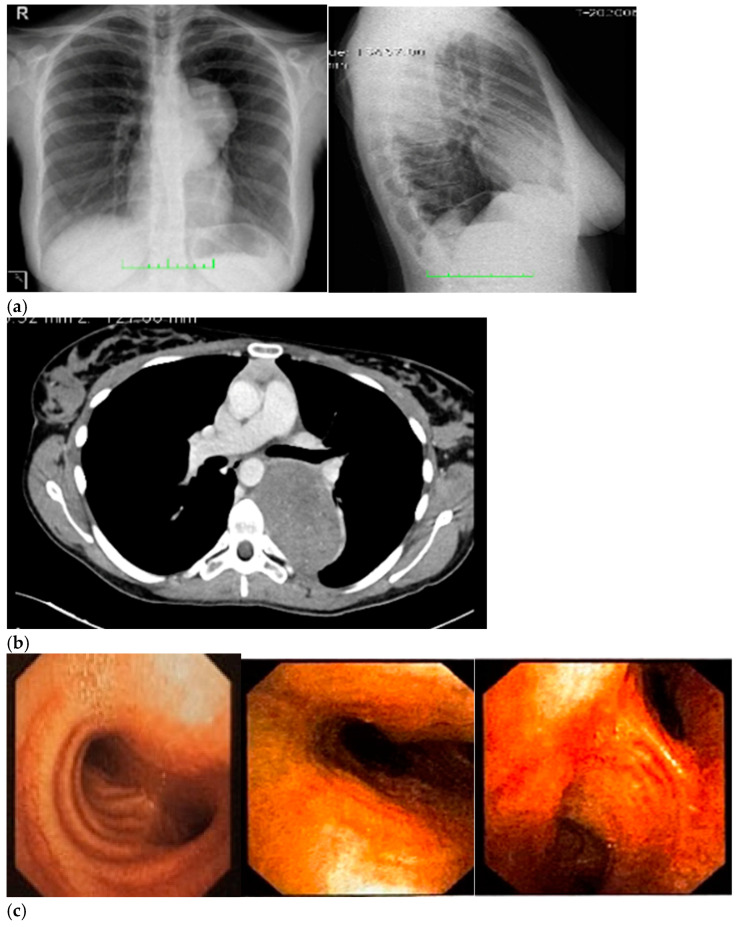

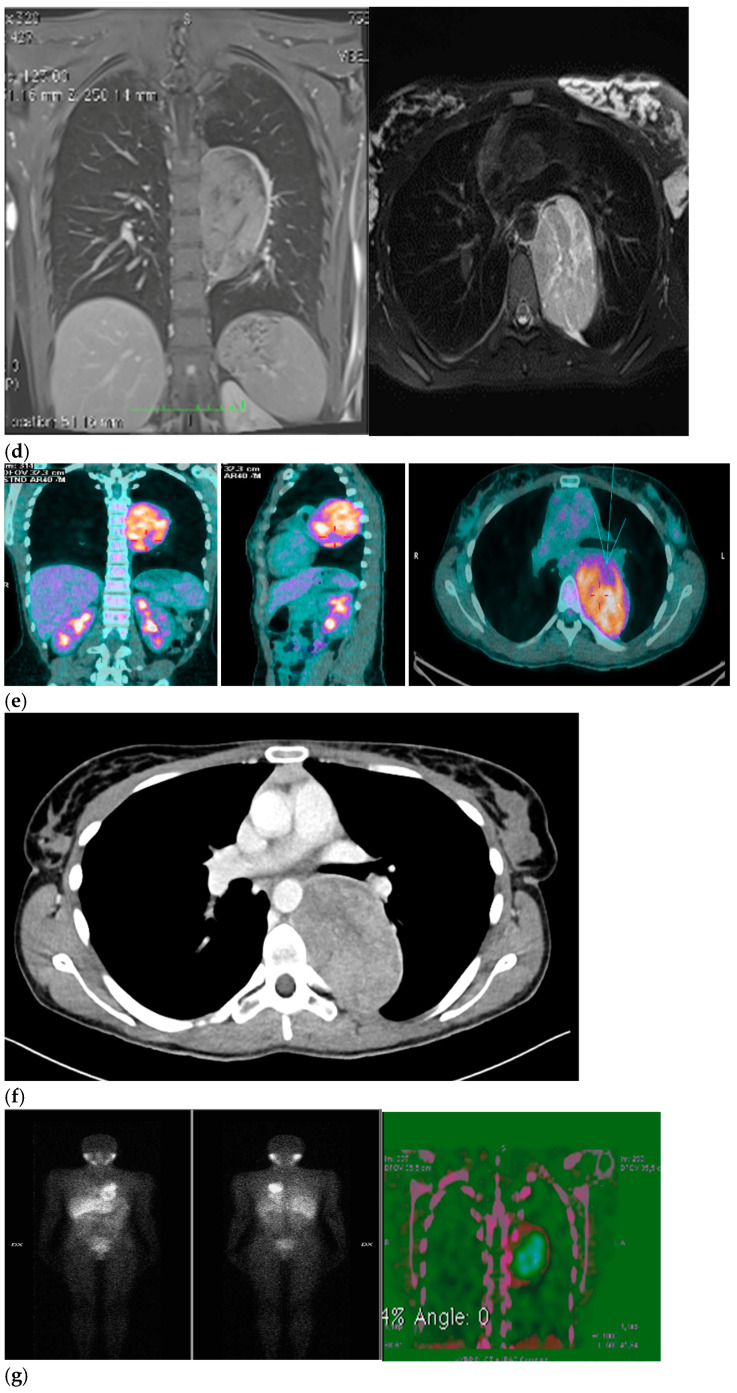

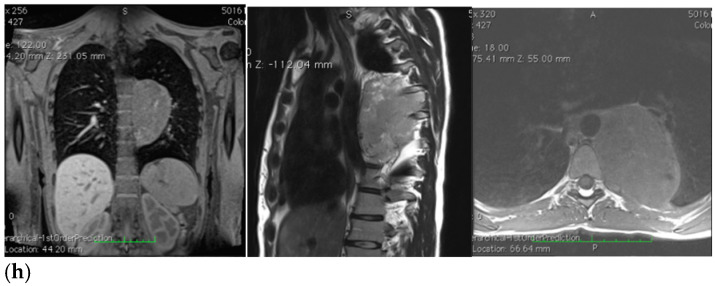
(**a**–**h**) Imaging assessments for pre-surgical evaluation. (**a**) X-ray scans. (NOTE: Voluminous opacity in the posterior mediastinum on the left, with a major axis of about 12 cm). (**b**) Thorax CT scan with Iodixanol. (NOTE: Solid, oval-shaped tumour in the left posterior mediastinum, measuring 8 × 5.5 cm. Anteriorly, the mass pressed the left main bronchus from behind, while medially it displaced the thoracic aorta—sitting directly on the metamers T5 to T8, and compressing the hemiazygos vein. The mass also went through the intervertebral foramen, between T6 and T7, and partially through the VI and VII intercostal spaces; laterally, it caused dystelectasis of the lung parenchyma. The NB had an irregular density, with innumerable small foci of calcification, and areas of slightly blurred impregnation of the contrast medium). (**c**) Tracheobronchoscopy. (NOTE: Left lower lobe bronchus with a luminal narrowing, resembling an extrinsic compression. In detail: left lower lobe bronchus, left main bronchus, trachea and carina). (**d**) Thorax MRI with Gadoteridol. (NOTE: Presence of a mass with maximum dimensions of approximately 7.5 × 5.5 cm axially and 10 cm of craniocaudal extension, limited by a plane passing from upper T6 to lower T9. Extension up to the ipsilateral lung, which caused a moderate compression and a slight compressive effect, also on the left sections of the heart (in particular the atrium). Slight anterior displacement of the ipsilateral pulmonary hilum structures; the neoplasm also came into contact with the aorta. The tumour went through the left T6–T7 intervertebral foramen—taking up space at the foraminal level, not at the level of the spinal canal—and it also extended up the corresponding intercostal space; it was compatible with a lesion originating from the nerve sheath arising from the T6–T7 foraminal region). (**e**) Total-Body PET/CT. (NOTE: Presence of an increased glucose metabolism lesion in the posterior mediastinal area, showing a necrotic portion and a metabolically more active portion, located in the medial side and infiltrating the posterior arch of the 6th left rib). (**f**) Thorax CT scan with Lopromide. (NOTE: Minor volumetric increase of the known space-occupying lesion, which maintained similar densitometric characteristics, clear limits, and maximum diameters equal to 8.5 × 6 cm on the transverse plane, with a maximum longitudinal extension of 11 cm. The solid component which went through the intercostal space between T6 and T7 was substantially unchanged. Absence of pleural or pericardial effusion). (**g**) Total-body scintigraphy with metaiodobenzylguanidine. (NOTE: The examination confirmed that the neoplasm was confined to the mediastinum only. The area of greater contrast medium enhancement corresponded to the left hemithorax, in particular, to the left posterior mediastinal region, from T6 to T9). (**h**) Thorax MRI with Gadoteridol. (NOTE: Slight increase in size of the known space-occupying lesion in the posterior mediastinum (85 × 60 × 103 mm vs. 80 × 55 × 100 mm). At T6–T7 level, the mass occupied some left foraminal canal space. Next to the known lesion, pleural effusion thin layer (maximum thickness of 11 mm)).

Therefore, her GP prescribed a chest X-ray, which revealed a voluminous opacity in her posterior mediastinum on the left (Figure 1a); subsequently, the GP referred the patient to a thoracic surgeon, who prescribed a series of imaging (Figure 1b–h) tests to biologically circumscribe the lesion, in order to evaluate its metabolic activity and its eventual malignancy. After these further investigations, the patient’s histopathological diagnosis was of poorly differentiated neuroblastoma ALK + (IIC), I stadium [16,17]. The patient was inserted in the operating room schedule for surgical excision.

The subsequent surgery revealed that the patient’s tumour was located only in the posterior mediastinum—between T6 and T9—without spinal canal involvement nor infiltration in either the organs or ribs. Furthermore, there was no evidence of cancer spreading or lymph node involvement. The patient was discharged 5 days after her surgery with a prescription for anticoagulants, antibiotics, and painkillers.

## 3. Results

The result of the definitive histological examination of the biopsy samples confirmed the diagnosis of poorly differentiated NB. Specifically, the NB was labelled as a malignancy with neuroectodermal differentiation (absent necrosis, 3 mitosis/10HPF), which showed a 10% proliferating fraction (Ki-67) that was positive for synaptophysin expression and focal for GFAP and protein S-100, while being negative for neurofilaments, EMA, cytokeratins, β-catenins, and CD99. The microscopic findings consisted of lobules of neuroblastic cells (Synaptophysin+), showing focal synchronous maturation of the nuclei and cytoplasm (maturing neuroblasts: <5%). These lobules appeared to be buried by fibrous septae, and populated by Schwann cells and blasts (S100+). Neuropil aggregates and focal calcifications were highlighted. The tumour appeared to be contained within surgical resection margins (1–10), with a low Mitosis-Karyorrhexis Index (MKI) (<100/5000 cells). The proliferation index of neoplastic cells (MIB1+) was 20%.

**Figure 2:**
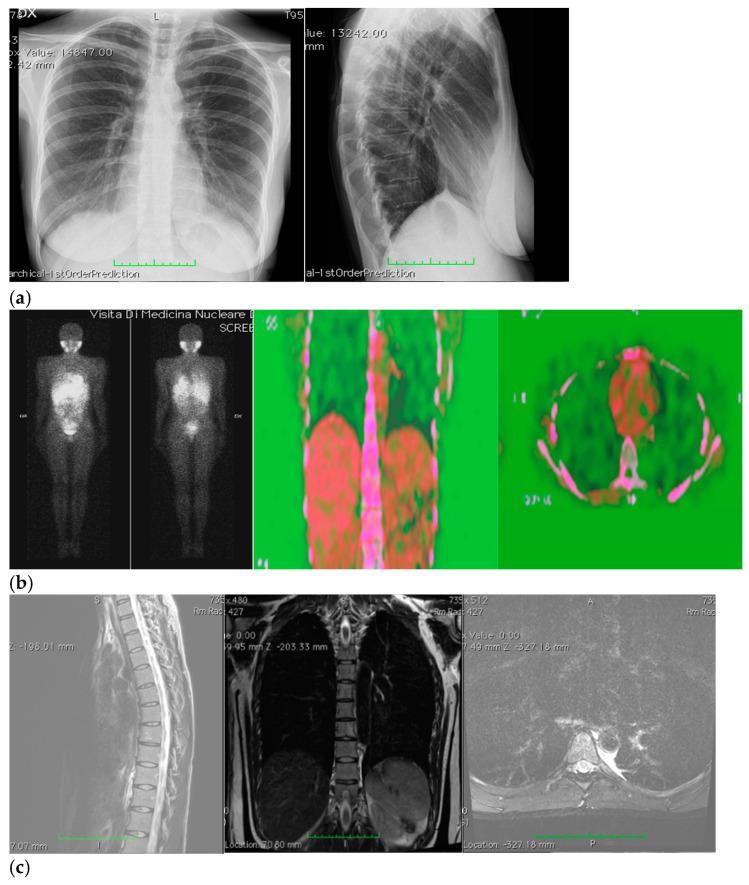
(**a**–**c**) Imaging assessments for post-surgical evaluation. (**a**) X-rays. (NOTE: No signs of ongoing pleuropulmonary lesions, free pulmonary bases, each hilum had a normal morphology, heart and aorta within normal limits, normal mediastinum). (**b**) Total-body scintigraphy with metaiodobenzylguanidine. (NOTE: No longer detectable pathological activity in the left hemithorax. No other areas of significant radiopharmaceutical were observed). (**c**) Thorax MRI with Gadoteridol. (NOTE: Outcomes of the surgical removal of the well-known left paravertebral lesion and a thin fluid layer corresponding to the surgical cavity. Very little slightly hyperintense remaining tissue in T2-weighted images, with subtle contrast enhancement and a maximum size of about 15 mm, at T6–T6 level, on the left side of the intervertebral foramen, close to the same level costovertebral joint—in a clinical picture which required MRI monitoring with contrast medium overtime).

Ten days after surgery, the patient stopped taking her pain medications and was able to sleep on her left side again; afterwards, her sutures were removed, and the patient underwent an imaging check-up, which had negative results (Figure 2).

Two months after surgery, her breast pain was still present, but it was intermittent and mild (2/10 NPRS). Her back pain, on the other hand, had a 4/10 NPRS intensity. It was intermittent, especially after more vigorous efforts, and more widespread; lastly, her tactile sensitivity under her left breast and on her left side surrounding area were absent.

Four months post-surgery, the patient went back to her physiotherapist to thank him for having “reported” her condition to her GP. She referred that her appetite was normal again, that she gained 4 kg following her surgical procedure, and that her back and breast pain were finally gone. The physiotherapist re-administered the Italian version of the SF-36, in order to assess the patient’s subjective health status (Table 2).

## 4. Discussion

Differential screening should be a physiotherapist’s mandatory skill in patients’ clinical managements, especially if they are under direct access. In this case report, through a comprehensive anamnesis and an appropriate physical examination, the physiotherapist detected a cluster of red flags suggesting that extra-professional competence could be required [31].

This highlights the necessity for physiotherapists to get advanced skills in collecting data, planning rational and targeted anamnestic research, and integrating data in an appropriate reasoning. Moreover, it could be necessary for health professionals to be trained in “inductive reasoning or pattern recognition”—which consists of formulating hypotheses after observing data and identifying some patterns attributable to specific pathological pictures and contributing factors [32]. Unfortunately, sometimes patients are referred to physiotherapists, but they are out of the physical therapy scope of practice [33,34,35]. For this reason, it is important for physiotherapists to perform a comprehensive anamnestic collection for each patient and to always screen for red flags, in order to understand if the patient must be treated, treated and referred, or referred [31].

Indeed, this not only implies the physiotherapist’s knowledge and skills required for interpreting the collected information type and quantity, but also calls for a particular ability in organizing and giving a clinical meaning to such data [31]. This could vitally reduce the time to diagnosis—a process to which many practitioners often contribute.

Furthermore, through a correct and careful medical history, the clinician may resort to some screening tests for more accurate and thorough management.

The physiotherapist has an ethical and moral duty to conduct differential screenings in order to rule out extra-expertise pathologies—both when the patient self-refers for rehabilitation assessment, and when they are referred by other practitioners. Unfortunately, it seems that physiotherapists with bachelor’s degrees might not have the specific tools and advanced training to conduct a proper anamnestic investigation, or to collect and integrate clinical data in order to correctly exclude pathologies that are beyond the scope of their expertise [36]. However, improving these skills should be crucial, since some clinicians may not be able to identify the symptom patterns underlying patients’—sometimes life-threatening—pathologies, or because a systemic or visceral picture may rise during the time between medical evaluation and physiotherapist consultation [37]. However, the prompt identification of signs and symptoms attributable to pathologies of medical interest, especially if they are severe, plays a key role in improving a patient’s clinical condition and life expectancy [37].

The anamnesis is an assessment milestone in the physiotherapist’s work: it contains almost all the required information to treat, treat and refer, or refer the patient directly to another health professional [37]; in this case report, numerous data collected during the anamnesis (Table 3) further contributed to reducing the likelihood of musculoskeletal diseases and strengthened the need for referral.

## 5. Conclusions

The diagnosis of NB in adults is a rare event [11]: the incidence rate for patients aged 20 to 39 years is approximately 0.2 cases per million in the United States [38]. This case report described an extremely rare case of NB, both for the patient’s age and for the unusual tumour histological behaviour and staging as poorly differentiated NB [3,6].

Probably, the tumour location and its slow growth rate made it possible for this predominantly paediatric cancer to take so long to manifest. Therefore, future research could focus on the biological mechanisms underlying the development of NB in adults, in order to manage and treat these types of patients according to specific guidelines. In fact, there are few reported cases of adulthood NB in the literature, and, consequently, the knowledge about this tumour’s implication in adult clinical manifestations is fairly restricted.

For these reasons, the physiotherapist—especially in direct access cases—must necessarily pay attention to all the findings from medical history and physical examination, which may be linked to an extra-professional pathology, and must be aware of how pathologies outside of his professional field can mimic musculoskeletal conditions. The greater the number of healthcare professionals involved in the patient’s management, the more communication and collaboration among them is important, with the ultimate goal of responding in the best possible way to the patient’s request for assistance.

Therefore, within their own professional field, each health practitioner must know how to act independently and observe professional boundaries, and do all this, at the same time, in a coordinated and synchronous manner with others.

In conclusion, the team-based nature of the diagnostic process oftentimes requires the contribution of many practitioners—who are able to face a patient’s “problem” through teamwork—with the aim of evaluating all possible scenarios.

## Figures and Tables

**Table 1:** Risk assessment and treatment strategies.

Pre-Treatment Classification System	Treatment Strategies	5-Years EFS	References
Very Low Risk Neuroblastoma (Stage L1/L2)	Clinical observation; Continuous monitoring through laboratory and instrumental investigations;	>85%	[22]
Low Risk Neuroblastoma (Stage L2-MS)	Chemotherapy cycles; Surgical resection; Radiotherapy;	>70–≤85%	[23]
Intermediate Risk Neuroblastoma (Stage L2-M-MS)	Variable chemotherapy cycles; Surgical resection;	≥50–≤75%	[24]
High Risk Neuroblastoma (Stage L1-L2-M-MS)	Four components High-dose chemotherapy; Primary tumour resection; Consolidation: myeloablative chemotherapy with reinfusion of autologous hematopoietic stem cells and external beam radiation theraphy; Immunotherapy with administration of Anti-GD2 Antibody Therapy, cytokines and cis-retinoic acid.	<50%	[25]

*Acronyms*: 5-years EFS, 5 years Event Free Survival.

**Table 2:** Clinical findings that emerged during the physical examination.

Assessment	Patient’s Results	
Vital parameters assessment: temperature, blood pressure and heart rate values	Normal (the temperature was 36.6 °C, the blood pressure was 124 systolic and 76 diastolic, and the heart rate was 72 beats per minute)	
Lungs auscultation	Negative: no rales nor other appreciable pathological sounds, average breathing rate.	
General observation: Observation of chest and thoracic cage	Negative: no swelling, ecchymosis, or bruising on her back or breasts, neither postural change.	
Active ROM of the dorsal spine and shoulders, in all directions	No active ROM abnormalities nor exacerbations of the painful symptoms	
Dorsal spine passive ROM	No passive ROM abnormalities nor exacerbations of the painful symptoms, but: thoracic spine passive movement in extension with overpressure made her back pain worse and her breast pain more acute; thoracic spine passive movement of anterior flexion with overpressure reduced her back and breast pain. However, the effect of these movements did not last long, and her symptoms returned to their usual intensity shortly after.	
Strength	Normal (5/5 Kendall’s scale): trunk extensor muscles, lateral trunk flexors, abdominal oblique muscles, upper abdominal muscles, and lower abdominal muscles	
Trigger points research in the patient’s back muscles	Normal: did not produce or modify the patient’s familiar pain	
Central and Unilateral Passive Accessory Intervertebral Motion tests	Pain was elicited and became deep and widespread in her left scapular area (7/10 NPRS)	
Postero-anterior Unilateral Passive Accessory Costo-Vertebral Motion tests on T5–T8	Pain was elicited and became deep and widespread in her left scapular area (7/10 NPRS)	
Supine Sign	Worse back pain (8/10 NPRS) and breast pain (7/10 NPRS)	
Closed-fist Percussion Sign	Worse back pain (8/10 NPRS) and breast pain (7/10 NPRS)	
Patient reported outcome measures, SF-36 for health-related quality of life *	Baseline	Follow-up
	PA = 50	PA = 95
	RP = 25	RP = 100
	BP = 22	BP = 84
	GH = 25	GH = 76
	VT = 10	VT = 70
	SF = 25	SF = 87
	RE = 0	RE = 100
	MH = 28	MH = 80

ACRONYMS: ROM, Range of Motion; NPRS, Numeric Pain Rating Scale; PA, Physical Activity; RP, Role Physical; BP, Bodily Pain; GH, General Health; VT, Vitality; SF, Social Functioning; RE, Role Emotional; MH, Mental Health. *, Range 0–100 (0 = less quality of life, 100 = better quality of life).

**Table 3:** Red flags that emerged during the anamnesis and physical examination.

Red Flags in the Patient’s Clinical Presentation	
Medical History	Physical Examination
Reported “bone pain” Constant pain Stabbing pain which increased with efforts Night pain, especially in supine position and on her left side Progressive onset Patient’s inability to evoke her familiar pain No trauma reported Probable weight loss Dyspnoea Wheezing Loss of appetite Asthenia Poor response to drugs History of neoplasm Family history of cancer	Positive Supine Sign Positive Closed-fist Percussion Sign Axial back pain Poor response to postural changes Painful passive accessory motion tests on T5–T8 vertebrae Symptoms which were not relieved by rest

## Data Availability

Not applicable.
